# Cross-language priming effects in bilingual novel metaphor processing

**DOI:** 10.1038/s41598-025-16841-5

**Published:** 2025-08-22

**Authors:** Katarzyna Jankowiak, Anna B. Cieślicka

**Affiliations:** 1 Faculty of English, Adam Mickiewicz University, Poznan, Grunwaldzka 6, Poznań, 60–780 Poland; 2 Department of Psychology and Communication, Texas A&M International University, Laredo, TX USA

**Keywords:** Novel metaphor, Cross-language priming, Bilingualism, ERPs, N400, LPC, Neuroscience, Cognitive neuroscience

## Abstract

This event-related potential study investigates whether semantically related words facilitate the processing of English novel metaphors and how this effect varies across within- and between-language contexts. Spanish-English/English-Spanish bilinguals performed a meaningfulness decision task in response to sentences in English, which included novel metaphors, novel similes, literal, and anomalous sentences. Prime words that were either related to the overall meaning of the target sentences or unrelated were presented either in English (the within-language condition) or Spanish (the between-language condition) before the sentence onset. In the N400 time window, unrelated primes evoked larger amplitudes than related primes in the within-language condition, indicating that conceptual compatibility reduces cognitive effort during early semantic processing. This effect was absent in the between-language condition, likely due to weaker associative links or additional cognitive demands from language switching. In the Late Positive Complex window, related primes imposed higher processing costs, reflecting their incorporation into sentence interpretation, while unrelated primes were dismissed earlier, reducing later-stage cognitive effort. These findings highlight the role of linguistic context in semantic processing, showing that within-language priming enhances efficiency, whereas between-language priming is less effective, particularly for complex novel metaphorical meanings.

## Introduction

Metaphor processing requires cross-domain mappings, which involves the recognition of shared features between two presumably distinct concepts^1,2^. The cognitive demands of cross-domain mappings are particularly pronounced when processing novel (i.e., highly unfamiliar and creative) metaphoric meanings (e.g., “Memory is a bag.”), where the metaphor source (i.e., *bag*) and metaphor target (i.e., *memory*) domains are weakly connected in semantic memory due to their infrequent co-activation^3^. As a result, a more robust activation in lexico-semantic memory is required to arrive at their interpretation^4,5^. Yet, the process of cross-domain mapping can be facilitated through contextual priming, where a preceding contextual cue (i.e., *capacious*) can facilitate lexical access and the construction of a meaning conveyed through a novel metaphor^6–8^. Little is known, however, whether and to what extent cross-domain mapping can be beneficial when contextual cues are presented in the same or a different language of a bilingual speaker. Therefore, the present event-related potential (ERP) study is the first to explore if new meaning construction could be facilitated when highly proficient bilingual speakers are presented with a prime cue in a within-language and a code-switched (i.e., between-language) condition.

Code-switching, defined as switching between languages, is a common phenomenon among bilinguals, and is especially prevalent among habitual code-switchers, who live in communities where code-switching is a commonplace and/or are used to frequent code-switching^9–11^. Despite extensive reaction time (RT) and ERP research indicating that code-switching is accompanied by considerable processing cost (e.g^12–14^.,, it is important to note that such effects appear to be reduced in habitual code-switchers (e.g^11,15,16^.,; see also the Adaptive Control Hypothesis^17^,. This indicates that in balanced bilinguals, who are proficient in both first (L1) and second (L2) language and code-switch regularly, lexico-semantic information is accessed prior to language membership information (see the Bilingual Interactive Activation Plus model, BIA+^18^; resulting in a preferential access of word meaning irrespective of the language in which the word is presented^19^.

Yet, code-switching is not only about switching between different language codes. Following the BIA + model^18^, it can be assumed that languages that are more active at a given moment (given the situational, linguistic, and task-driven contexts), attain a higher resting level state, similar to those that are more dominant for a given bilingual speaker. In the case of habitual code-switchers, both languages maintain similar levels of activation, facilitating their efficient selection during code-switching. The robust co-activation of both languages in habitual code-switchers might result in a more automatic spreading of activation between the two language systems. Consequently, balanced bilinguals may benefit from more extensive spreading activation mechanisms in their lexico-semantic network during both language production and comprehension. This is evident in phenomena such as between-language priming effects (i.e., L1–L2 or L2–L1), demonstrating that the two languages can effectively prime one another when their resting level states are comparably high^20–22^.

In the context of ERPs, priming effects are usually reflected in the modulations of the two ERP components: the N400 and the Late Positive Complex (LPC). The N400, observed at centro-parietal electrode positions and peaking around 400 ms after stimulus onset, is widely associated with lexico-semantic access^23^. Research employing the priming paradigm has consistently reported smaller N400 amplitudes in response to semantically or associatively related primes, indicating facilitated processing (e.g^24–26^.,; for a discussion see^27^. In contrast, the LPC, typically emerging around 600–800 ms post-stimulus and often maximal over posterior regions, has been linked to processes such as memory updating, reanalysis, and controlled aspects of meaning integration^28,29^. In priming paradigms, LPC modulation has been interpreted as reflecting the evaluation of stimulus congruity or the recruitment of explicit memory processes, particularly when semantic relationships are less automatic or more strategic. Here, our aim is to test the strength of within- as compared to between-language priming within an experimental setting where spreading activation mechanisms are cognitively demanding, as they involve cross-domain mappings engaged in novel metaphor processing. By situating the current study within this ERP literature, we aim to clarify the functional contributions of the N400 and LPC to the processing of metaphorical meaning across languages, and to better understand the cognitive mechanisms underlying metaphor comprehension in bilingual contexts.

Priming is assumed to occur as a result of the automatic spreading of activation between related features^30,31^. In novel metaphor processing, which is hypothesized to encompass structural alignment of the components of the literal and metaphorical meaning^32,33^, primes often involve a word that is semantically related to the literal meaning of a target metaphor (e.g^6–8^.,. For instance, in a cross-modal study by^7^, prime words including *plant* and *spike* facilitated the processing of the subsequent target metaphor *John doesn’t like physical contact. Even his girlfriend finds it difficult to come close to him. John is a cactus.* In the context of ERP studies, such semantically related, compared to semantically unrelated, primes have been shown to facilitate lexico-semantic processing of metaphors, as reflected in reduced amplitudes of the N400^8^, an ERP component marking lexico-semantic processing^23^. Here, we aimed to test if a word semantically related to the correct reading of a metaphor can prime its subsequent processing (e.g., *aggressive* | *These lawyers are sharks*.), and whether such a facilitation, reflected in the N400 modulations, could be equally demonstrated in both the within (e.g., L1–L1) and between-language contexts (e.g., L2–L1).

Crosslinguistic priming, defined as the facilitation of processing in one language by prior exposure to semantically related material in another language, provides crucial evidence for the interconnectedness of bilinguals’ lexico-semantic systems (e.g^34,35^.,. Research has demonstrated that bilinguals can experience semantic priming effects not only within a single language but also across their two languages, suggesting that activation of conceptual representations can spread independently of language membership. For instance, in crosslinguistic priming, reduced N400 amplitudes for translation equivalents or semantically related cross-language primes have been observed (e.g^20,22^.,, thus supporting the notion that conceptual representations are shared across languages and that conceptual access precedes language-specific selection^18^. In the context of novel metaphor processing, where robust conceptual mapping is necessary, crosslinguistic priming may similarly facilitate comprehension by allowing bilingual speakers to draw on a shared conceptual system regardless of the language in which the prime is presented. Thus, investigating crosslinguistic priming effects provides insights that help understand how bilinguals leverage their integrated lexico-semantic resources to support complex meaning construction across languages.

Importantly, while previous monolingual studies employing a priming paradigm in metaphor research (e.g^7,8,36^., have largely examined primes related to the literal source domain of the metaphor (e.g., “John is a cactus.” being primed by “plant” or “spike”^7^;, our focus is on whether semantic priming of the figurative interpretation can similarly facilitate metaphor comprehension. This shift allows us to explore how quickly and efficiently readers access the intended figurative meaning, particularly in the case of novel metaphors, where literal interpretations may be misleading or less informative. We argue that examining figuratively related primes captures a different, yet complementary, aspect of metaphor processing by more directly tapping into the successful interpretation of metaphorical meaning. This approach also aligns with theoretical accounts that emphasize the importance of conceptual mappings and feature alignment in arriving at a metaphor’s intended interpretation^34,38^.

The present study aims to investigate whether priming facilitates novel metaphor processing in bilinguals and whether this facilitation is modulated by the language of the prime. To this end, we presented Spanish-English/English-Spanish bilinguals with English novel metaphors, novel similes, literal, and anomalous sentences preceded by related or unrelated prime words in either the within-language condition (i.e., English primes) or between-language condition (i.e., Spanish primes) to examine the strength of L1–L1 and L2–L1 priming effects. Specifically, we hypothesized reduced N400 amplitudes for related than unrelated primes both in the within- and between-language priming conditions, reflecting effective spreading activation across their lexico-semantic networks and heightened co-activation of both languages in habitual code-switchers. In addition to the N400, this study also examines the Late Positive Complex (LPC), an ERP component associated with final meaning integration and reanalysis. The LPC has been widely investigated in previous ERP research on bilingual processing, including novel metaphor comprehension^4,5^. By including both the N400 and LPC, this study provides novel insights into the interplay between bilingualism, code-switching, and novel metaphor processing, advancing our understanding of the cognitive and neural mechanisms underlying bilingual language processing.

## Methods

### Participants

Forty-four Spanish-English/English-Spanish bilinguals participated in the experiment. Data sets from 19 participants were discarded due to either the low quality of the EEG recordings (*N* = 7), failure to meet the accuracy threshold of 70% in the behavioral data (*N* = 7), or both (*N* = 5). The final pool reported here consisted of 23 participants (6 male, 17 female, *M*_*age*_ = 22.6, *SD* = 3.63, range: 19–33). All participants had normal/corrected-to-normal vision and no language or neurological disorders. They were students at a large Hispanic-serving US institution, and they volunteered to take part in the study for extra credit.

Language History Questionnaire (LHQ3^38^; was used to collect participants’ language background data. Fifteen participants reported Spanish as their native language (henceforth L1) and 8 declared English as their L1. Over half of the participants (*N* = 14) were early bilinguals, acquiring both languages prior to 5 years of age. Out of the 15 L1- Spanish participants, 4 were born in Mexico, one in Puerto Rico, and the remaining 10 were born and raised in the US. Two participants reported country of residence as Mexico, with the remaining 21 residing in the US.

L1-English bilinguals were overall more proficient in English than Spanish, across all four language domains (listening: *p* <.01; speaking: *p* <.01; reading: *p* <.05; and writing: *p* <.001). On the other hand, proficiency scores for L1-Spanish bilinguals were highly comparable for both English and Spanish across all the usage domains, except writing, which was scored higher for English (*M* = 6.0, *SD* = 1.71) than for Spanish (*M* = 4.79, *SD* = 1.67, *p* <.05). L1-Spanish bilinguals rated their Spanish speaking proficiency (*M* = 5.71, *SD* = 1.49) and Spanish writing proficiency (*M* = 4.79, *SD* = 1.67) significantly higher than L1- English bilinguals (Spanish speaking proficiency: *M* = 3.83, *SD* = 1.47, *p* <.05; Spanish writing proficiency: *M* = 2.83, *SD* = 1.94, *p* <.05) (see Table 1 for summary of the participant characteristics).

In addition, participants’ dominance score was calculated based on the LHQ3 aggregated dominance scores and the L2 to L1 dominance ratio. Bilinguals with a ratio below 0.7 (range 0.28–1.96) were categorized as L1-dominant, those with a score between 0.7 and 1.4 were categorized as balanced, and bilinguals with a score above 1.4 were classified as L2-dominant. Thirteen bilinguals were dominant in English and 10 were balanced users of both languages.

**Table 1:** Participants’ language background information.

	L1 English (*N* = 8)		L1 Spanish (*N* = 15)	
Dominance	Eng-dom (*N* = 7)		Eng-dom (*N* = 6)	
	Span-dom (*N* = 0)		Span-dom (*N* = 0)	
	Balanced (*N* = 1)		Balanced (*N* = 9)	
AoA Spanish	Early (0–5 years of age) (*N* = 5) Late (after 5 yoa) (*N* = 3)			
AoA English			Early (0–5 years of age) (*N* = 9) Late (after 5 yoa) (*N* = 6)	
	**L1 English**		**L1 Spanish**	
	**English**	**Spanish**	**English**	**Spanish**
Listening	6.83 (0.41)**	5.50 (1.38)	6.36 (1.15)	6.36 (1.34)
Speaking	6.67 (0.52)**	3.83 (1.47)*	6.07 (1.27)	5.71 (1.49)
Reading	6.83 (0.41)*	4.00 (2.0)	6.29 (0.1)	5.57 (1.83)
Writing	6.83 (0.41)**	2.83(1.94)*	6.0(1.71)*	4.79 (1.67)

Proficiency ratings in various language dimensions were measured with a 7-point Likert scale where 1 = very poor, 7 = native like. Asterisks show significant differences in proficiency ratings for English and Spanish. Values for proficiency include mean rating and SD in parentheses.

* *p* <.05, ** *p* <.01, *** *p* <.001.

### Stimuli

#### Target sentences

The target sentences included 480 declarative and emotionally-neutral English sentences divided into four categories: 120 novel metaphors (e.g., *My heart is a* ***drawer***.; *Bacteria are* ***fighters***.; *Motivation is an* ***engine***.*)*, 120 novel similes (e.g., *My heart is like a* ***drawer***.; *Bacteria are like* ***fighters***.; *Motivation is like an* ***engine***.*)*, 120 literal sentences (e.g., *This piece of furniture is a* ***drawer***.; *These boxers are* ***fighters***.; *This machine is an* ***engine***.*)*, and 120 anomalous sentences (e.g., *A bug is a* ***drawer***.; *Gifts are* ***fighters***.; *A frog is an* ***engine***.). The linguistic stimuli were adopted from a database by^39^ that provides a set of pre-tested novel metaphors, novel similes, literal, and anomalous sentences. The stimuli were highly controlled for their levels of meaningfulness, familiarity, and metaphoricity by means of conducting norming tests on English native speakers. Furthermore, critical (sentence-final) words were all concrete nouns, and were controlled for their frequency (SUBTLEX-US^40,41^; *M* = 3.93, *SD* = 0.56), number of letters (*M* = 6.57, *SD =* 1.45) and syllables (*M* = 2.34, *SD* = 0.48)^39^.

#### Prime words

For each target sentence, a set of four prime words was developed, creating four possible priming conditions: (1) English related, where an English word was related to the sentence meaning and facilitated its interpretation; (2) English unrelated, where an English word was not related to the sentence meaning; (3) Spanish related (a translation equivalent of the English related prime word) (4) Spanish unrelated (a translation equivalent of the English unrelated prime word). Effort was made to create primes that would be conceptually related to the overall meaning of the metaphorical or literal sentence, rather than associatively related to only its last word. Since a proper comprehension of a novel figurative expression requires mapping semantic features from the source onto target domain^42^ or from the vehicle to its topic^43^ with the aim of establishing a metaphor’s ground, we assumed that by presenting a word priming the expression’s ground, the resulting interpretation of the subsequent novel metaphor or novel simile would be easier. English primes whose translations were cognates or inter-language homographs were replaced with synonyms, such as the final set of primes did not include any cognates nor cross-language homographs (see Table 2 for examples of experimental stimuli in each condition). Half of the prime words were nouns, and the other half – adjectives.

**Table 2:** Sample stimulus set, along with english/spanish related and unrelated primes.

Sentence type	Experimental sentence	Priming condition			
		English related	English unrelated	Spanish related	Spanish unrelated
Novel Metaphor	Motivation is an engine	STRENGTH	BUN	FORTALEZA	BOLLO
Novel simile	Viruses are like travelers	BOUNDLESS	FOGGY	DESMESURADOS	BRUMOSOS
Literal	These enclosures are cages	JAILED	WISE	ENCARCELADO	SABIO
Anomalous	An elbow is sugar	TASTY	MARRIED	RICO	CASADO

Overall, the experimental stimuli included 120 sentences in each category (novel metaphors, novel similes, literal sentences, anomalous sentences) paired with 480 primes (120 English-related, 120 English-unrelated, 120 Spanish-related, 120 Spanish-unrelated). In addition, 240 anomalous filler sentences were created, half of which were paired with unrelated English (e.g., *This test was a piece of sausage- SKILL*) and half with Spanish unrelated words (*The tea is too nervous- ENLAZADO* [Eng. “linked”]).

To fully counterbalance the four prime types with the four types of experimental sentences, four experimental lists were created. Each list consisted of 120 sentences in each category, for the total of 480 experimental sentences such that within each list each sentence occurred once but with a different prime type. Within each category, half of the sentences (*N* = 60) were paired with English prime words and half (*N* = 60) with Spanish prime words. Half of the English/Spanish primes within each sentence category (*N* = 30) were related to the meaning of the target sentence and the other half (*N* = 30) were unrelated. In addition to the 480 experimental sentences, each list included 240 anomalous fillers, half of which (*N* = 120) were paired with unrelated English and the other half (*N* = 120) with unrelated Spanish words.

#### Stimuli norming

English primes and their Spanish translations were normed for concreteness, frequency, number of letters, and number of syllables (see Table 3 for summary of stimuli characteristics). As is typical for Spanish and English translations, Spanish targets were longer in terms of the number of letters (*p* <.001) and syllables *(p* <.001) than English ones. Word frequencies were selected from the SUBTLEX-ESP database^44^ for Spanish and SUBTLEX-US^40^ for English. Concreteness ratings were obtained from^45^ for English and from^46^ for Spanish. Because of an effort to avoid using interlanguage homographs or cognates, many translations had to be replaced with less frequent Spanish synonyms. As a result, Spanish primes, both related and unrelated had a lower frequency *(p* <.001) and concreteness *(p* <.001) than English ones. It is to be noted though that concreteness data had many missing values on account of the Spanish concreteness databases being limited. Therefore, the dimension of concreteness needs to be treated with caution as the presented average might not be reflective of all the words in the stimulus dataset.

**Table 3:** Means for concreteness, number of letters, number of syllables, and word frequency for english and Spanish prime target words. Square brackets represent SE.

	English primes		Spanish primes	
	Related	Unrelated	Related	Unrelated
Frequency ^1^	3.78 [0.04]	4.18 [0.04]	2.47 [0.04]	2.73 [0.04]
Concreteness ^2^	3.78 [0.04]	3.61 [0.05]	2.24 [0.07]	2.80 [0.07]
Number of letters	6.57 [0.09]	6.18 [0.09]	7.39 [0.10]	6.92 [0.10]
Number of syllables	1.93 [0.04]	1.88 [0.03]	3.16 [0.04]	2.95 [0.04]

^1^Based on SUBTLEX-US and SUBTLEX-ESP: log frequency.

^2^Based on concreteness ratings from^45^ for Spanish: 1- very abstract, 9- very concrete; English concreteness values based on^44^ – the Spanish scale was recoded to be comparable with English: 1-abstract, 5-concrete.

To ensure that the experimental sentences are appropriate for the bilingual population being tested, two norming surveys were prepared: a familiarity norming and prime-relatedness norming. The norming surveys were prepared and distributed using the *Qualtrix XM Platform*. Eighty-two Spanish-English/English-Spanish participants were recruited to complete the online surveys. The survey participants shared the same background as the experimental pool, but none of them took part in the subsequent experiment.

In the familiarity norming survey, participants were presented with experimental sentences and asked to rate how frequently they see or hear each sentence on a scale from 1 to 7 (1: I never hear/see the sentence; 7: I hear/see the sentence very often). Twenty-one participants were recruited to complete the familiarity norming survey. *ANOVA* conducted on the mean ratings obtained for experimental sentences revealed a significant main effect of Sentence Type (*F*(3, 60) = 29.3, *p* <.001, η^2^_p_ = 0.60). Anomalous sentences were rated significantly lower (*M* = 1.39, 95% CI [1.01, 1.78]) than novel metaphors (*M* = 2.40, 95% CI [1.98, 2.82]), *p* <.001, literal sentences (*M* = 3.23, 95% CI [2.64, 3.82]), *p* <.001, and novel similes (*M* = 2.50, 95% CI [2.07, 2.93]), *p* <.001. In addition, both metaphor (*M* = 2.40, 95% CI [1.98, 2.82]) and novel simile ratings (*M* = 2.50, 95% CI [2.07, 2.93]) were significantly lower than the ratings obtained for literal sentences (*M* = 3.23, 95% CI [2.64, 3.82]), *p* <.001.

In the prime-relatedness norming survey, participants were presented with experimental sentences paired with English/Spanish related or unrelated words and instructed to rate how much the word paired with the sentence is related on a scale from 1 to 7 (1: totally unrelated, 7: totally related). Given that the overall number of experimental sentences (*N* = 480) paired with primes in the four conditions was very large (*N* = 1932), six surveys were prepared, each including *N* = 322 sentences. Each survey included 80 sentences from each category (novel metaphors, novel similes, literal sentences, anomalous sentences), 20 paired with English-related, 20 with English-unrelated, 20 with Spanish-related and 20 with Spanish-unrelated primes. Sixty-one participants completed the prime-relatedness norming survey.

Results of the survey were entered into a three-way *ANOVA* (Sentence Type: Novel metaphor vs. Novel simile vs. Literal vs. Anomalous x Prime Relatedness: Related vs. Unrelated x Language: English vs. Spanish). The analysis revealed significant main effects of the Sentence Type (*F*(2, 122) = 10.54, *p* <.001, *η*^*2*^*p* = 0.15), Relatedness (*F*(1,61) = 290.49, *p* <.001, *η*^*2*^*p* = 0.83), and Language (*F*(1, 61) = 11.60, *p* =.001, *η*^*2*^*p* = 0.16), as well as a significant two-way interaction between Sentence Type and Relatedness (*F*(2, 122) = 35.11, *p* <.001, *η*^*2*^*p* = 0.37), and Relatedness by Language (*F*(1, 61) = 8.53, *p* =.005, *η*^*2*^*p* = 0.12). Related primes were rated significantly higher (*M* = 4.86, 95% CI [4.67, 5.05]) than unrelated primes (*M* = 2.47, 95% CI [2.22, 2.72]), (*p* <.001). Overall, English primes obtained a higher mean rating (*M* = 3.74, 95% CI [3.58, 3.91]) than Spanish primes (*M* = 3.59, 95% CI [3.40, 3.77]), (*p* =.001). Relatedness by Language interaction revealed that English related primes were rated significantly higher (*M* = 5.00, 95% CI [4.80, 5.19]) than English unrelated primes (*M* = 2.49, 95% CI [2.24, 2.74]), (*p* <.001). Likewise, Spanish related primes were rated significantly higher (*M* = 4.72, 95% CI [4.52, 4.93]) than Spanish unrelated primes (*M* = 2.45, 95% CI [2.20, 2.71]), (*p* <.001). Moreover, English related primes (*M* = 5.00, 95% CI [4.80, 5.19]) elicited higher ratings than Spanish related primes (*M* = 4.72, 95% CI [4.52, 4.93]), (*p* <.005). Sentence by Relatedness interaction showed that English related primes accompanying literal sentences were rated higher (*M* = 5.31, 95% CI [5.11, 5.52]) than English related primes for novel metaphors (*M* = 4.75, 95% CI [4.55, 4.94]), (*p* <.001).

### Procedure

The study was conducted in accordance with the Belmont Report. All the methods and procedures applied in the experiment were approved by the Institutional Review Board at Texas A&M International University (Protocol No.  #2023-03-03, approved on 24 March 2023). Written informed consents were obtained from all participants involved in the study. EEG data recording was conducted in a sound-attenuated room. Participants were seated at approximately 70 cm away from a 21-inch LCD monitor (Dell OptiPlex 7000 SFF computer, Intel Core i7-12700 Processor; OS Windows 11 Pro). Experimental sentences were presented in E-Prime 2.0 (Psychology Software Tools Inc.).

Each experimental trial started with a focusing cross displayed for 1000 ms. After the focusing cross disappeared, a prime word was presented centrally in black font (Verdana 18, bold) on a gray opaque background. The prime duration was 400 ms. Following the prime, a target sentence, up to the penultimate word, was displayed left-aligned for 1200 ms. Duration of the prime and sentence display was determined in trial test runs with participant volunteers recruited from the same population as the experimental participants. Volunteer participants provided feedback as to whether they had sufficient time to read and interpret the prime and the sentence, so the timing adjustments were made accordingly. Following the first part of the sentence, the penultimate word of the sentence was displayed centrally for 600 ms, followed by the presentation of the last critical (target) word. The last word remained on the screen until the participant responded, but no longer than 4,000 ms.

Participants performed a meaningfulness decision task, whereby they were asked to decide whether the sentence displayed on a computer screen was meaningful or meaningless. They were told that they would see an English or Spanish word presented briefly before each sentence and instructed not to focus on the word, but to read each sentence carefully, trying to understand its meaning. Participants were advised that once they saw the penultimate and final words displayed centrally, they should refrain from blinking and excessive movement and prepare to make their decision whether the sentence was meaningful or not. Upon seeing the final word, they pressed the corresponding button on the Chronos Response Box to indicate their decision. To minimize movements, participants were instructed to rest their hand on the Chronos Response box throughout the experiment. Response buttons were counterbalanced across participants. Prior to the experimental session, participants completed a practice block to get familiarized with the task. Given the large number of experimental sentences, the session was divided into four blocks with rest pauses in between. The rest pauses were under participants’ control, and they could take as much time as needed to relax their muscles and rest the eyes. Each block consisted of 180 trials (30 novel metaphors, 30 novel similes, 30 literal sentences, 30 anomalous sentences) and 60 anomalous fillers. The experimental session lasted about 70 min. Once the EEG recording was finished, participants completed the LHQ3 questionnaire.

### EEG data recording

EEG data were recorded at 2048 Hz from 64 Ag/AgCl electrodes placed at the standard extended 10–20 positions. The bipolar electrodes monitoring vertical (vEOG) and horizontal (hEOG) eye movements were placed above and below the left eye and next to the outer rim of the right eye, respectively. The EEG signals were recorded by ActiView (Biosemi) and amplified using an ActiveTwo AD-box (Biosemi). Since the recommendations for the BioSemi ActiveTwo system suggest recording an EEG signal with an impedance level for each electrode of no more than ± 50 µV, in the present experiment, we ensured that the impedance (offset) level for each electrode was kept at ± 20 µV.

### Behavioral data analysis

Reaction Times (RTs) smaller than 200 ms or exceeding 3 standard deviations above the mean were discarded (2.8% of the data) and analyzed with a linear mixed-effects model (LMM) using the R studio (R Core Team, 2023). A linear mixed-effects model was fit using restricted maximum likelihood estimation model^47^. The following fixed effects were included: language (English, Spanish), relatedness (related, unrelated), sentence type (novel metaphor, novel simile, literal, anomalous), and their interactions. Because the participants differed in their Spanish proficiency and because of frequency differences between Spanish and English primes, both Spanish proficiency and prime word frequency were included as covariates. The fixed effects were sum-coded. Maximal models with a full random-effect structure were first computed, with by-subject and by-item random intercepts, the fixed effects of language, relatedness, sentence type and their interactions as by-subject and by-item random slopes, and with Spanish proficiency and prime frequency as covariates. The maximal model did not converge. We therefore proceeded to reduce the complexity of the model incrementally, removing the least informative slope until the model converged. The selected optimal model was based on Akaike Information Criterion (AIC)^48,49^, under which lower values indicate a better fit. The final model included random intercepts for subjects and items, and by-subject random slopes for sentence type. This model showed the best fit (AIC = 283475.7). *P*-values were calculated using Satterthwaite approximations for degrees of freedom. The outcomes of the final fitted model are provided in the OSF: https://osf.io/vkag6/.

### Electrophysiological data analysis

We analyzed two ERP components previously reported to be modulated by metaphoricity level and language of operation^4,5^: the N400 and LPC. The ERP analyses were performed within pre-defined time windows: 300–500 ms (N400) and 600–800 ms (LPC) over the FC1, FCz, FC2 (fronto-central), C1, Cz, C2 (central), CP1, CPz, CP2 (centro-parietal), and P1, Pz, P2 (parietal) electrodes.

BrainVision Analyzer 2.1 software (Brain Products, Germany) was used to analyze the data offline. Continuous EEG data were down-sampled to 500 Hz, re-referenced to the common average reference^48,49^ and filtered offline (Butterworth zero phase filters) with a high-pass filter set at 0.1 Hz (slope 24 dB/octave) and a low-pass filter set at 20 Hz (slope 24 dB/octave). They were then segmented from 200 ms before critical word onset to 1500 ms afterward, baseline-corrected relative to signal between − 200 and 0 ms before stimulus^1^ (i.e., critical word) onset, and edited for artifacts (i.e., rejecting trials with flatlining events, voltage differences higher than 100 µV, or voltage steps higher than 50 µV). Ocular artifacts were corrected using the ocular artefact regression method^53^. As a result of artifact rejection procedures, 2.83% of trials with novel metaphors, 1.20% with novel similes, 1.74% with literal sentences, and 2.03% with anomalous sentences were excluded.

ERPs were time-locked to the onset of the critical word of each sentence, which was placed in a sentence-final position. Within both the N400 and LPC time frames, mean ERP amplitudes were analyzed employing repeated measures *ANOVA* analyses, with 2 (Priming language: Within-language vs. Between-language) × 2 (Prime-Sentence relatedness: Related vs. Unrelated) × 4 (Sentence type: Literal sentences vs. Novel similes vs. Novel metaphors vs. Anomalous sentences) as within-subject factors. Anterior-posterior electrode position (frontocentral vs. central vs. centro-parietal electrodes vs. parietal) along with Laterality (left vs. midline vs. right electrodes) were included in the analyses as within-subject factors. The Greenhouse-Geisser correction was applied when the sphericity assumption was violated, as indicated by the Mauchly’s tests. Pairwise comparisons were corrected for multiple comparisons with the Bonferroni correction.

## Results

### Behavioral data

#### Reaction times

**Fig. 1:**
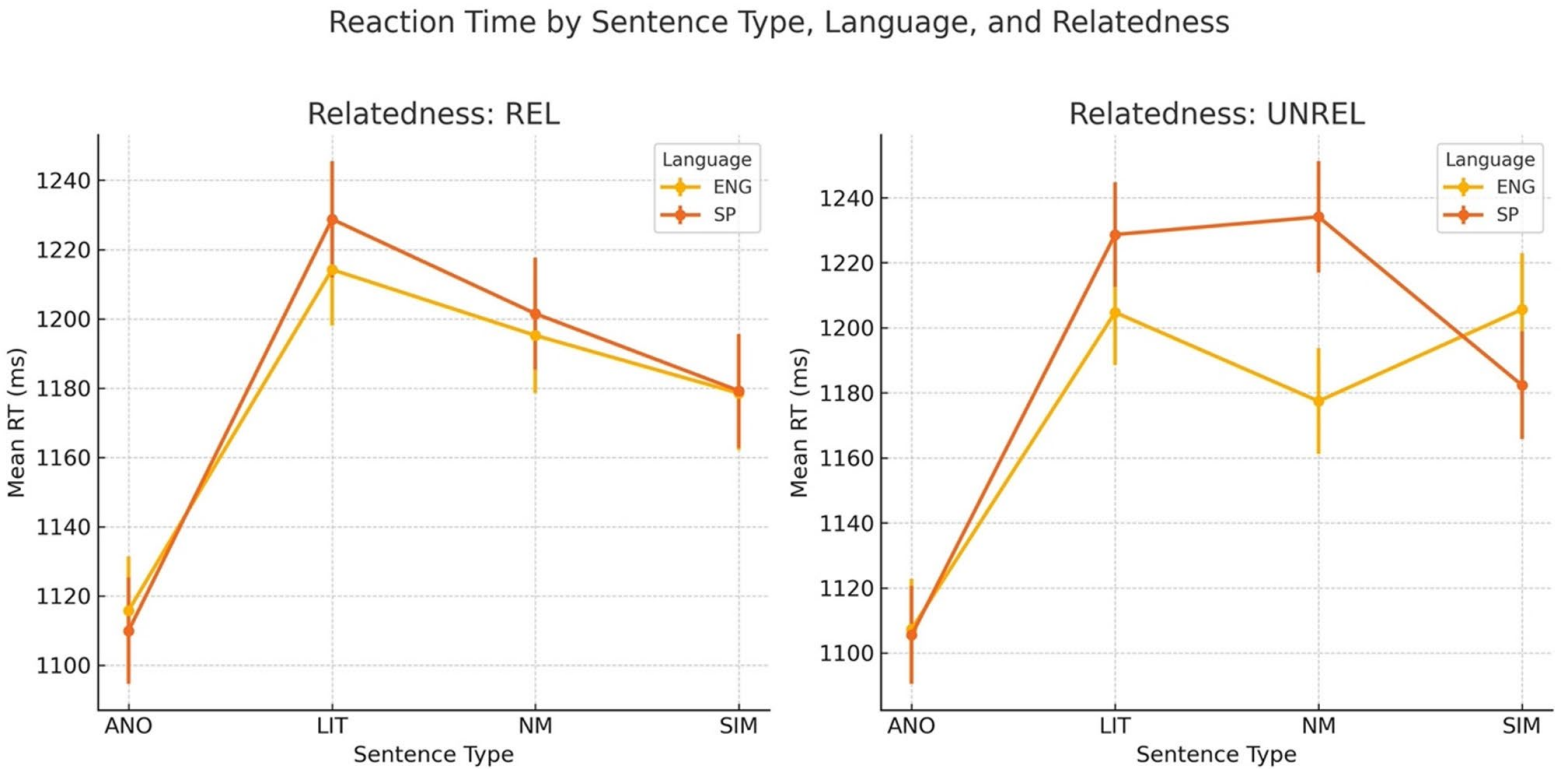
RT (Reaction time) data in milliseconds showing time for the meaningfulness decision task in response to anomalous (ANO), literal sentences (LIT), novel metaphors (NM), and novel similes (SIM), preceded by related (Rel) and Unrelated (Unrel), Spanish (Sp) and English (Eng) primes. Bars represent SE (Standard Error).

The analysis showed a significant main effect of Sentence Type [*F* = 35.54, *p* <.001, *η*^*2*^ = 0.005]. Reaction times were significantly slower for literal sentences compared to anomalous ones (*t* = 9.54, *p* <.001, *β* = 0.19), for novel metaphors compared to anomalous (*t* = 8.06, *p* <.001, *β* = 0.16), and for novel similes compared to anomalous (*t* = 6.70, *p* <.001, *β* = 0.13). No significant main effects were found for Language [*F* = 1.18, *p* =.278, *η*^*2*^ = 0.000] or Prime Relatedness [*F* = 0.12, *p* =.73, *η*^*2*^ = 0.000], and none of the interactions were significant (*p*s > 0.20). Spanish proficiency and prime frequency were also non-significant predictors. These results indicate that anomalous sentences were significantly easier to make a meaningfulness judgment about than the remaining three sentence types, regardless of language or semantic relatedness of the prime. No significant differences were observed between the sentence types themselves (all *p* >.05), although there was a trend for literal sentences to yield slower RTs than novel similes (*p* =.061) (see Table 4; Fig. 1 for summary of the RT data).

**Table 4:** Mean RTs elicited for the four sentence types paired with related and unrelated english and Spanish primes. Square brackets represent SE.

Sentence type	English		Spanish	
	Related	Unrelated	Related	Unrelated
Novel metaphor	1196 (16.7)	1175 (16.8)	1209 (18.3)	1236 (16.6)
Literal	1213 (17.1)	1202 (16.8)	1231 (18.2)	1232 (17.2)
Novel simile	1180 (16.6)	1205 (16.7)	1182 (17.7)	1182 (16.8)
Anomalous	1115 (17.2)	1110 (16.8)	1100 (18.2)	1110 (18.1)

#### Accuracy rates

To obtain a full picture of how the different sentence types were processed, we also analyzed participants’ responses to the meaningfulness judgment task. The expected responses would be homogenous for anomalous sentences, which were all semantically incorrect and hence called for the “meaningless”/No response and for literal sentences, which were all semantically correct and hence called for the “meaningful”/Yes response. Provided the participants understood the intended figurative meanings of novel similes and novel metaphors, they should also judge them as meaningful, although the responses here might differ, depending on an individual’s perception and degree of their figurative competence.

Analysis of participants’ responses revealed that, as expected, anomalous sentences were the easiest to make a correct decision about, with 93% of correct responses. Next, literal sentences elicited 65% of the correct responses, followed by novel similes, where only 45% of the participants judged them to be meaningful. Finally, novel metaphors were assessed as meaningful by only 41% of the respondents, suggesting they posed most difficulty. A one-way *ANOVA* conducted on the responses revealed that these differences in the rates of correct responses between sentence types were statistically significant. Specifically, anomalous sentences obtained a significantly higher number of correct responses (M = 0.93) than literal sentences (*M* = 0.65; *t*(36.1), *p* <.001), novel similes (*M* = 0.45; *t*(60)), *p* <.001), and novel metaphors (*M* = 0.41; *t*(66), *p* <.001). Literal sentences were recognized as meaningful significantly more than either novel metaphors (*t*(24.8), *p* <.001) or novel similes (*t*(20.55), *p* <.001). Finally, novel similes were recognized as meaningful significantly more often than novel metaphors (*t*(4.06), *p* <.001).

### Electrophysiological data

#### N400 (300–500 ms)

**Fig. 2:**
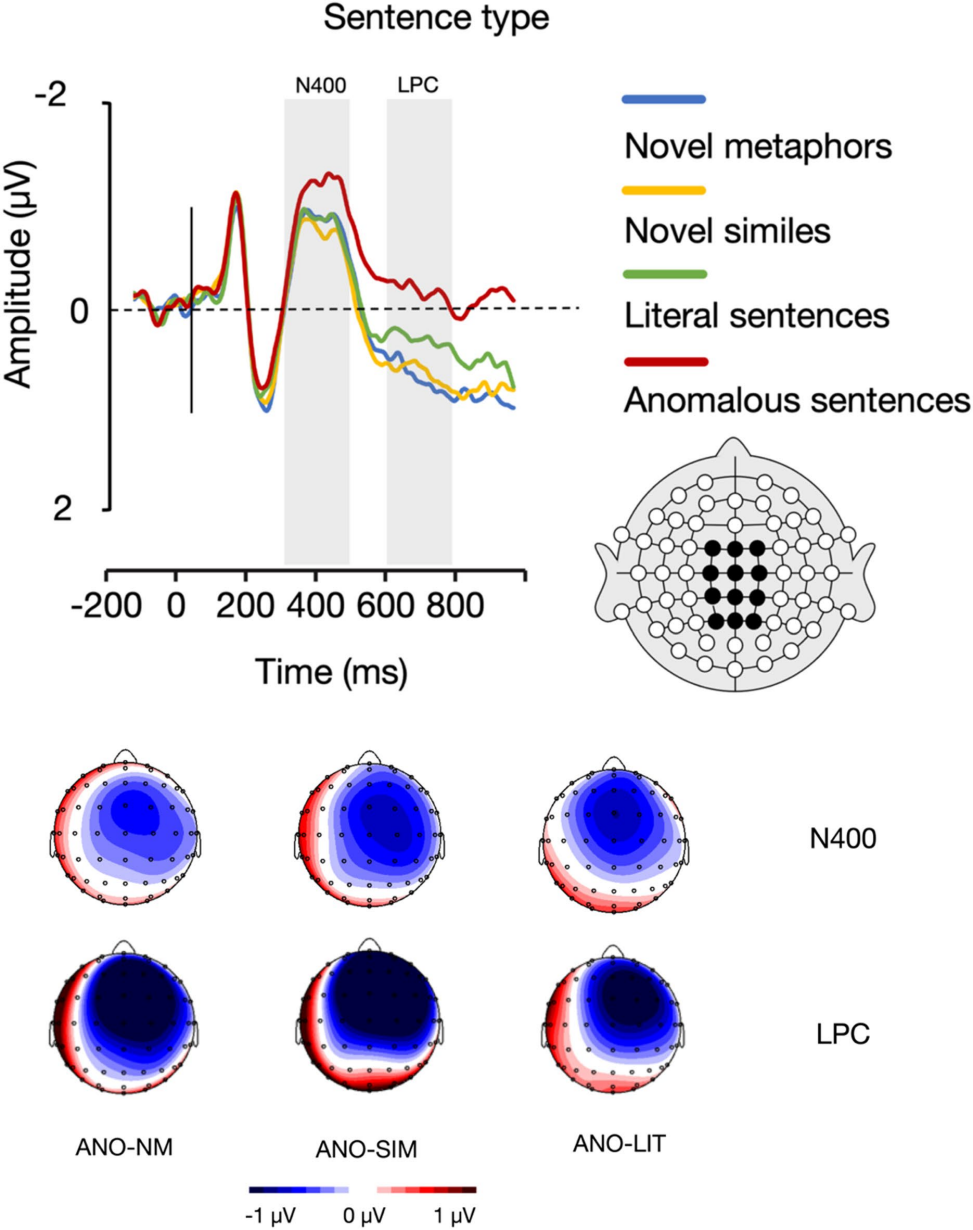
A visual representation of the main effect of sentence type in the N400 (300–500 ms) and LPC (600–800 ms) time windows. The grand average waveforms represent the mean activity across twelve electrodes. The topographic maps illustrate the difference waves between anomalous sentences (ANO) and novel metaphors (NM), novel similes (SIM), and literal sentences (LIT), as observed in the N400 and LPC time windows.

Within the N400 time window (300–500 ms), the analysis of variance (*ANOVA)* showed a main effect of sentence type (*F*(3, 66) = 3.93, *p* =.012, η_p_^2^ = 0.152). Bonferroni-corrected pairwise comparisons further showed that anomalous sentences (*M* = − 0.94 µv, *SE* = 0.22) elicited significantly larger N400 amplitudes than novel similes (*M* = − 0.49 µv, *SE* = 0.27), *p* =.039, and literal sentences (*M* = − 0.58 µv, *SE* = 0.20), *p* =.035. There were no statistically significant differences between anomalous sentences and novel metaphors (*M* = − 0.60 µv, *SE* = 0.21), nor between novel metaphors and both novel similes and literal sentences, and between novel similes and literal sentences, *p*s > 0.05 (Fig. 2).

**Fig. 3:**
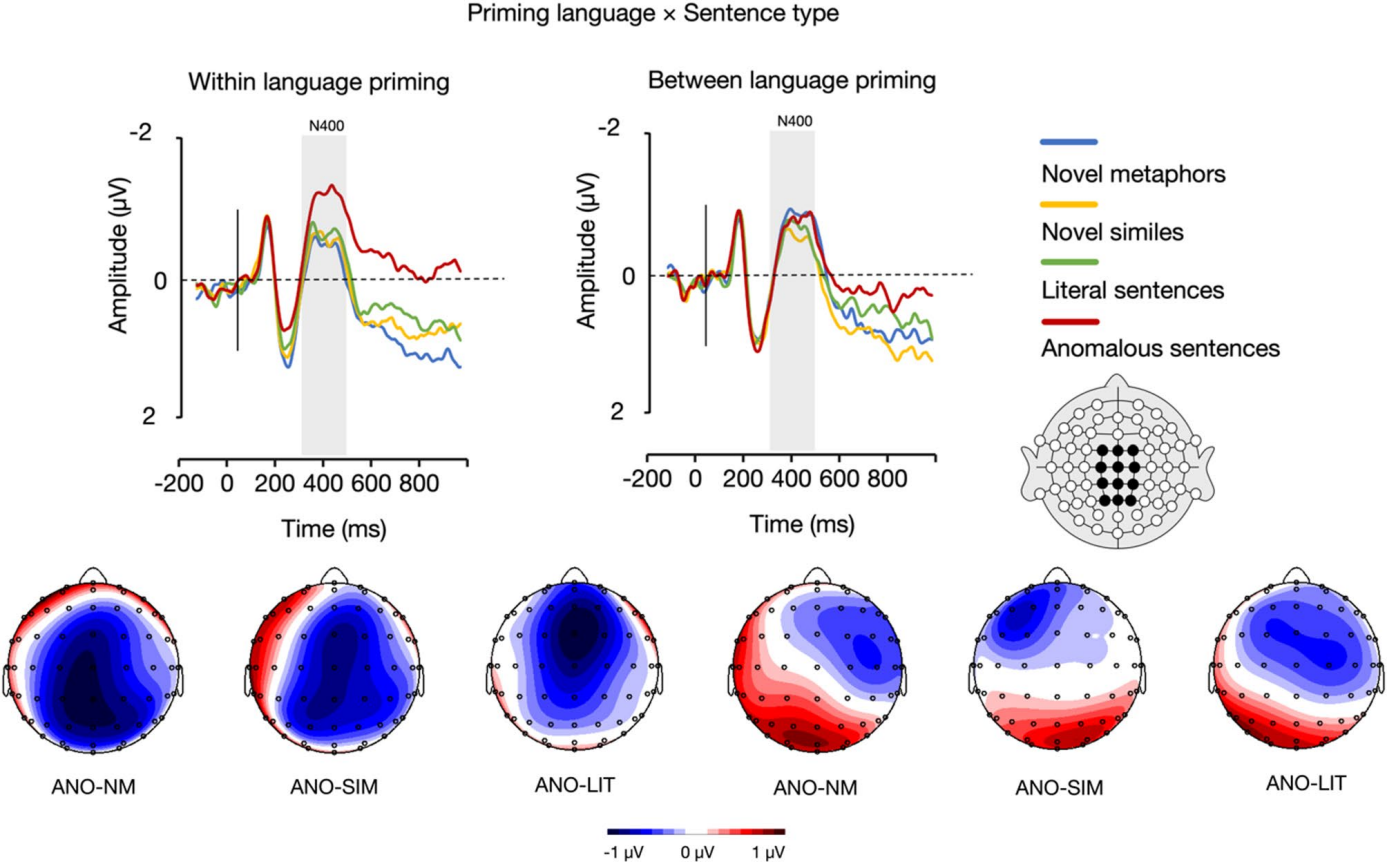
A visual representation of the interaction between priming language and sentence type in the N400 (300–500 ms) time window. The grand average waveforms represent the mean activity across twelve electrodes. The topographic maps illustrate the difference waves between anomalous sentences (ANO) and novel metaphors (NM), novel similes (SIM), and literal sentences (LIT), separately for within and between language priming, as observed in the N400 time window.

Then, the analysis yielded an interaction between priming language and sentence type (*F*(3, 66) = 3.49, *p* =.020, η_p_^2^ = 0.137). Post-hoc analyses were conducted separately for English (within-language priming) and Spanish (between-language priming). In the case of English primes, anomalous sentences (*M* = −1.18 µv, *SE* = 0.24) elicited significantly larger N400 amplitudes than novel similes (*M* = − 0.53 µv, *SE* = 0.30), *p* =.024, literal sentences (*M* = − 0.63 µv, *SE* = 0.18), *p* =.049, and novel metaphors (*M* = − 0.42 µv, *SE* = 0.22), *p* =.019. There were no statistically significant differences between novel metaphors and both novel similes and literal sentences, nor between novel similes and literal sentences, *p*s > 0.05. In the case of Spanish primes, there were no statistically significant differences between any of the sentence types, *p*s > 0.05 (Fig. 3).

**Fig. 4:**
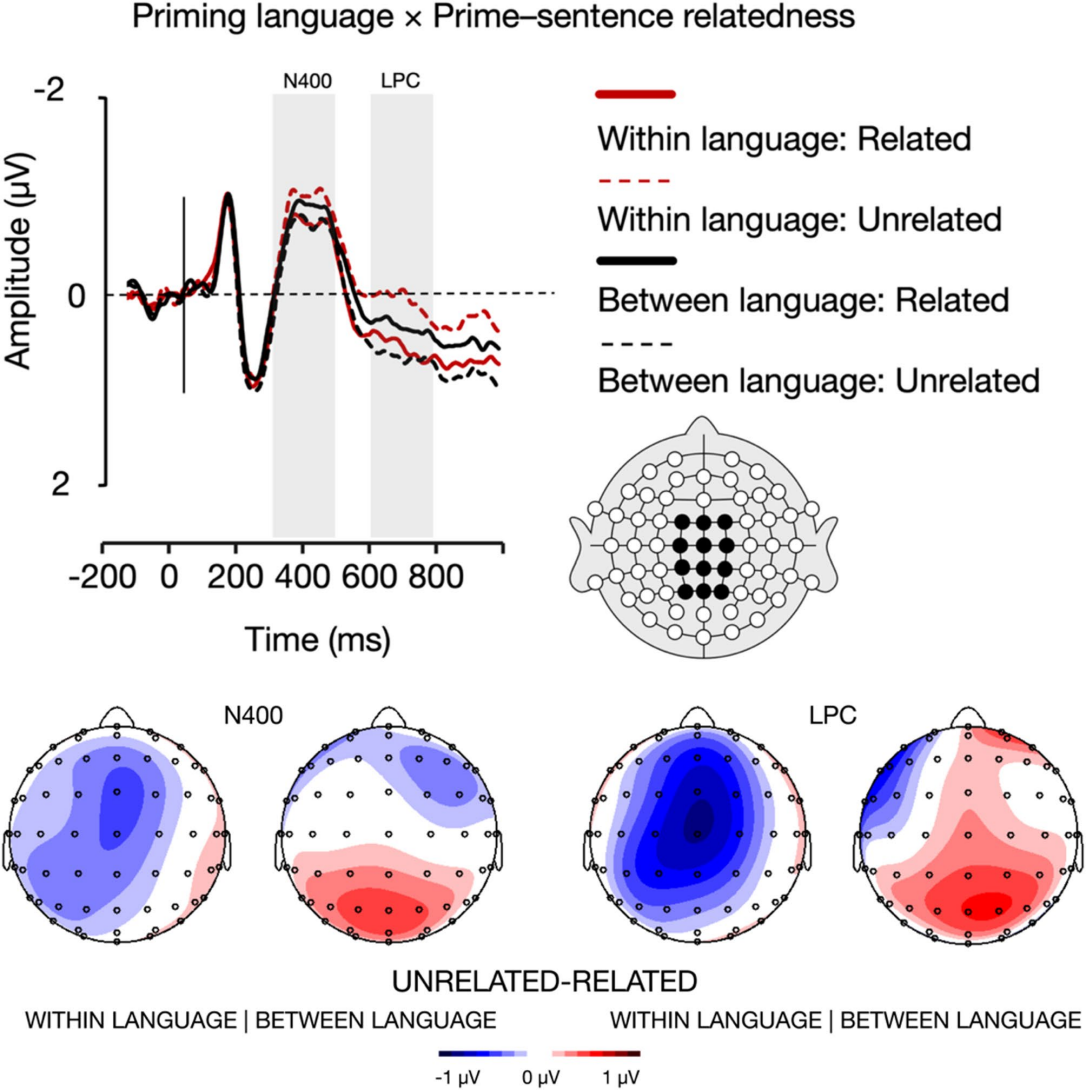
A visual representation of the interaction between priming language and prime–sentence relatedness in the N400 (300–500 ms) and LPC (600–800 ms) time windows. The grand average waveforms represent the mean activity across twelve electrodes. The topographic maps illustrate the difference waves between unrelated and related conditions, separately for within and between language priming, as observed in the N400 and LPC time windows.

Finally, the results showed a marginally significant interaction between priming language and prime-sentence relatedness (*F*(1, 22) = 3.99, *p* =.058, η_p_^2^ = 0.154). Bonferroni-corrected pairwise comparisons further showed a trend, whereby for sentences following English primes (i.e., the within-language priming condition), the unrelated condition (*M* = − 0.82 µv, *SE* = 0.20) evoked larger N400 amplitudes than the related condition (*M* = − 0.56 µv, *SE* = 0.24), *p* =.097. No such trend was observed for sentences following Spanish primes (i.e., between-language priming), *p* =.277 (Fig. 4).

#### Late positive complex (LPC; 600–800 ms)

Within the LPC time window (600–800 ms), the analysis of variance (*ANOVA)* showed a main effect of sentence type (*F*(3, 66) = 8.73, *p* <.001, η_p_^2^ = 0.284). Bonferroni-corrected pairwise comparisons further showed that anomalous sentences (*M* = − 0.02 µv, *SE* = 0.28) elicited significantly smaller LPC amplitudes than novel metaphors (*M* = 0.80 µv, *SE* = 0.37), *p* =.010, and novel similes (*M* = 0.76 µv, *SE* = 0.31), *p* =.005. There were no statistically significant differences between anomalous sentences and literal sentences (*M* = 0.44 µv, *SE* = 0.25), nor between novel metaphors and both novel similes and literal sentences, and between novel similes and literal sentences, *p*s > 0.05 (Fig. 2).

In addition, the analysis yielded an interaction between anterior-posterior electrode position and sentence type (*F*(9, 198) = 8.56, *p* <.001, η_p_^2^ = 0.280). Post-hoc analyses further showed that statistically significant differences between sentence types were observed only among fronto-central and central electrode positions, whereby anomalous sentences elicited significantly smaller LPC amplitudes than novel similes (FC: *p* <.001, C: *p* =.002) than literal sentences (FC: *p* =.028, C: *p* =.049), and novel metaphors (FC: *p* =.002, C: *p* =.012).

Finally, the results showed an interaction between priming language and prime-sentence relatedness (*F*(1, 22) = 6.65, *p* =.017, η_p_^2^ = 0.232). Bonferroni-corrected pairwise comparisons further showed that for sentences following English primes (i.e., within-language priming), the unrelated condition (*M* = 0.17 µv, *SE* = 0.21) evoked smaller LPC amplitudes than the related condition (*M* = 0.64 µv, *SE* = 0.30), *p* =.048. No such trend was observed for sentences following Spanish primes (i.e., between-language priming), *p* =.148 (Fig. 4).

## Discussion

The present ERP study aimed to test if a word semantically related to the correct reading of a metaphor can prime its subsequent processing, and whether such a facilitation is further modulated by the within or between-language context. To this end, Spanish-English/English-Spanish bilinguals performed a meaningfulness decision task involving English novel metaphors, novel similes, literal, and anomalous sentences that were primed by a related or unrelated word presented in either English (a within-language condition) or Spanish (a between-language condition).

The behavioral data did not show any relatedness nor language effects, in that participants’ responses were unaffected by whether the target sentence was preceded by English or Spanish prime and whether the prime was related or unrelated to the sentence. However, sentence type significantly affected participants’ responses, such that anomalous sentences elicited shorter response times than the remaining sentence types, regardless of the language and relatedness of the prime. These results indicate that participants found anomalous sentences easy to reject as semantically meaningless. This is to be expected, given that semantic decomposition of an anomaly very early on indicates violation of the semantic constraints and hence allows making a fast meaningfulness decision. On the other hand, no such violation is present in literal sentences, which therefore must be fully processed and interpreted before making a meaningfulness decision. Finally, novel metaphors and novel similes require additional cognitive effort to extract semantic features from the source and target domains and evaluating such features for their correspondences. This ultimately leads to an increased processing effort, as reflected in the longer RTs that were obtained in the meaningfulness decision task.

ERP results revealed distinct effects of sentence type in both the N400 and LPC time windows. During the N400 window, anomalous sentences elicited significantly larger amplitudes than both novel similes and literal sentences, reflecting increased lexico-semantic processing difficulty. This aligns with prior bilingualism research on metaphor processing^4,5^ and supports the view that semantic incongruity heightens cognitive effort^23^. In contrast, the LPC window showed the opposite pattern: anomalous sentences elicited smaller amplitudes than novel metaphors and novel similes. This may reflect the overlap of a sustained negativity, commonly associated with semantic conflict^53,54^ with the LPC component. The reduced LPC amplitude for anomalous sentences likely indicates limited engagement in semantic reanalysis due to their lack of meaningful content. By contrast, the enhanced LPC for novel metaphors and similes suggests increased cognitive effort in integrating unfamiliar figurative meanings, consistent with the view that the LPC indexes late-stage semantic reprocessing and integration^55–58^.

Importantly, these neural dynamics must be discussed in light of the task demands. Participants were explicitly instructed to perform a meaningfulness judgment task, requiring them to decide as quickly and accurately as possible whether each sentence was meaningful. This task recruits both automatic and controlled semantic processes. Accordingly, early ERP components such as the N400 reflect automatic lexico-semantic access and mismatch detection, especially when faced with semantically anomalous stimuli. In contrast, later components such as the LPC index more controlled, effortful reanalysis and integration, particularly when interpreting novel or ambiguous figurative expressions. Thus, the observed ERP patterns highlight a key distinction in processing strategy: anomalous sentences evoke early semantic conflict (N400) followed by disengagement from deeper analysis (reduced LPC), whereas novel metaphors and novel similes prompt extended semantic processing and creative integration (enhanced LPC), despite less pronounced early negativity. The absence of sustained negativity for novel metaphors may further suggest more efficient or context-supported processing.

Interestingly, this neural dissociation corresponds only partially with the behavioral findings. While anomalous sentences were behaviorally the easiest to reject, as reflected in the fastest response times and highest accuracy, their more pronounced N400 responses reveal significant early processing costs. In turn, the behavioral and neuropsychological data for novel metaphors and similes aligned, in that these expressions were behaviorally more challenging, as manifested in longer RTs and lower response accuracy, and they also elicited larger LPC amplitudes, indicative of increased cognitive engagement and sustained semantic evaluation. This divergence between the mismatching early vs. late EEG and behavioral data underscores the importance of considering both behavioral and neural measures; while behavioral data capture decision outcomes, ERP data illuminate the underlying processing dynamics. Together, they reveal the complexity of bilingual figurative language processing as shaped by sentence type, processing stage, and task context.

Furthermore, the results revealed an interaction between priming language and sentence type within the N400 time frame, whereby anomalous sentences evoked larger N400 amplitudes relative to all the other conditions, yet only in within-language priming. This interaction suggests that within-language priming facilitates a more robust sensitivity to semantic anomalies during early lexico-semantic processing, as indexed by the N400 response. Specifically, anomalous sentences eliciting larger N400 amplitudes in the within-language condition indicate heightened difficulty in integrating semantically incongruent information when the prime and target sentence are in the same language. In the within-language priming condition, the semantic network is likely pre-activated more effectively due to the shared linguistic context, which enhances the ability to detect mismatches between the prime and the target sentence^59^. As a result, when the target sentence is anomalous, it triggers a stronger N400 response, reflecting greater cognitive effort required to resolve the semantic incongruity^23^. This heightened response is absent in the between-language priming condition, possibly because cross-language priming engages semantic representations less efficiently^60^ or introduces additional processing demands through language-switching costs^61,62^, thereby dampening sensitivity to the anomaly. Altogether, the larger N400 amplitudes for anomalous sentences in within-language priming highlight the role of shared linguistic context in amplifying semantic mismatch detection, while the absence of this effect in between-language priming suggests the attenuated efficiency of cross-language semantic integration.

The N400 results showing priming differences for anomalous vs. novel similes, novel metaphors, and literal sentences might have also originated from a different nature of the primes that these sentences were paired with. As previously specified, the priming words paired with metaphorical and literal sentences were conceptually related (e.g., *A shopaholic is a hunter*: ***goal;*** *Adoption is like a wedding*: ***loyalty***) as the intention was to enable faster and more efficient mappings between the source and target domains that are required for a successful interpretation of a novel figurative expression. On the other hand, because anomalous sentences by default do not lend themselves to a conceptual prime, the primes created for anomalous sentences were lexical level, related associatively to the last word of the sentence (e.g., *That potato is a kidnapper*: ***hijack***). It is possible that the associative nature of primes for anomalous sentences affected the subsequent sentence processing differently than was the case with conceptual-level primes that were used for the remaining sentence types.

Finally, the ERP results showed an interaction between priming language and prime-sentence relatedness within the time windows of both the N400 and LPC. In the N400 time frame, sentences preceded by unrelated primes evoked larger N400 amplitudes compared to those preceded by related words, yet only in the within-language priming condition. Such a priming effect, in contrast, was not observed in the between-language priming condition. On the other hand, in the LPC time window, sentences preceded by unrelated primes evoked smaller LPC amplitudes compared to those preceded by related words, with the effect being again observed only in the within-language priming condition.

In the N400 time frame, the facilitated processing of sentences preceded by related primes in the within-language priming condition suggests that conceptual compatibility between the prime and the target sentence plays a critical role in reducing cognitive effort during early semantic processing^63,64^. Related primes appear to pre-activate shared semantic networks, streamlining the lexico-semantic access required for sentence comprehension^55,56^. The absence of this effect in the between-language condition may indicate that cross-language priming fails to engage overlapping semantic representations as efficiently, potentially due to weaker associative links or the added cognitive demand of switching between languages^67,68^. This finding aligns with bilingual models of language processing, such as the Revised Hierarchical Model^69^, which propose that cross-language activation is less automatic and requires additional processing resources, especially when semantic connections are less direct.

In the LPC time frame, the observed pattern points to a different cognitive mechanism at play. The more cognitively taxing processing of sentences preceded by related primes, compared to unrelated primes, suggests that the prime’s relevance to the sentence interpretation introduces additional integration demands. Specifically, the semantic compatibility of the related prime may necessitate its incorporation into the final interpretation of the sentence, thereby imposing a higher processing cost during the later stages of meaning integration. By contrast, unrelated primes are likely dismissed as irrelevant early in processing, reducing the need for further cognitive effort at this stage. This interpretation aligns with the role of the LPC in reflecting extended cognitive mechanisms such as reanalysis, elaboration, and integration of complex or competing semantic information^55,56^.

Lack of between-language priming in the results reported here might also be related to the participants’ language experience. Specifically, our participant pool consisted of both L1 Spanish and L1 English bilinguals, highly proficient in English. All but one L1-English participants and 6 out of 15 L1-Spanish bilinguals were dominant in English, with the remaining bilinguals categorized as balanced. More importantly, all were students in an English-speaking institution whose reading and writing proficiency scores were higher in English than in Spanish. The all used English daily for academic (reading and writing) purposes, while Spanish was mostly used in conversations with friends and family members. Therefore, the visual nature of the primes in the current design might not be as effective for Spanish as it was for English, the language that our bilingual participants predominantly read and write in. A cross-modal design, with primes presented auditorily, would perhaps be a better indicator of the degree to which between-language primes facilitate metaphorical sentence comprehension.

Furthermore, the absence of between language priming effects observed in the current study may be attributable to several methodological factors. First, the timing of the stimulus onset asynchrony (SoA) may not have been optimal for capturing priming effects across languages. Second, differences in participants’ familiarity with primes in the two languages could have affected the results, particularly if primes in one language were less familiar to participants, thereby diminishing their activation strength. While no effect was found for prime frequency in the behavioral analysis, regardless of items frequency, our bilinguals’ individual experiences with Spanish primes might have differed. Norming the primes on subjective familiarity, in addition to frequency, seems to be a desired step in future studies. Third, the relatively limited sample size may have constrained statistical power, reducing the likelihood of detecting subtle or marginal cross-language effects. Future studies should consider systematically manipulating SoA timing, carefully matching lexical frequency across languages, and increasing sample sizes to more reliably assess the presence or absence of cross-language priming.

Differences in the electrophysiological responses to sentences preceded by English vs. Spanish primes may, in part, reflect the brain’s sensitivity to code-switching. Prior research has shown that mixed-language input can elicit distinct ERP responses, particularly in the N400 and LPC time windows^12,16,70–73^. The N400 has been linked to semantic integration difficulty during language switching^74–77^, while the LPC is commonly interpreted as a neural marker of code-switching, indexing late-stage integration or reanalysis processes^15,71^. Recent findings suggest, however, that the N400 is not reliably elicited by code-switches when they are contextually and semantically expected. In contrast, LPC effects tend to emerge regardless of contextual fit, reflecting the general processing cost of language switching^71,78–80^. For instance^71^, found that English-Spanish bilinguals did not exhibit an N400 effect when encountering semantically predictable code-switches (e.g., *He put a clean sheet on the cama* [bed]), yet these same code-switches elicited robust LPC effects. Similarly^80^, demonstrated that N400 effects were tied to semantic fit rather than language switching per se, whereas LPC effects persisted across switch conditions.

Still, direct comparison between these findings and our study is limited due to crucial methodological differences. Most ERP studies on code-switching involve sentence-internal or final switches that are syntactically integrated and constitute translation equivalents of expected words (e.g^11,78,81^.,. These designs allow the code-switched element to become meaningfully embedded in the sentence. In contrast, our primes were single Spanish words presented before the English sentence, with no syntactic or semantic incorporation into the target sentence. This structural disconnect may have disrupted any potential facilitation expected from the prime, particularly if the processing cost of the code-switch itself overshadowed the benefits of semantic priming.

Participant background may also have influenced these effects. Code-switching costs have been shown to vary depending on habitual switching behavior, with habitual or dense code-switchers often incurring lower cognitive costs^70,78,79^. Yet such benefits may be restricted to contexts where switching is intentional and contextually supported. Our participants came from a bilingual border community where dense, often unintentional switching is the norm. The isolated, pre-sentential Spanish prime (detached from the syntactic and pragmatic flow of the English sentence) may therefore have conflicted with their naturalistic switching habits, contributing to greater processing cost rather than facilitation.

Furthermore, asymmetries in switch direction must be considered. Prior studies have shown that switches from the dominant to the non-dominant language (e.g., English to Spanish) tend to be more taxing than the reverse^15,74^. Given that most of our participants were balanced or English-dominant, switching from Spanish to English should have been relatively manageable. Yet any potential ease may have been offset by the timing and structure of the prime presentation. The relevance of the prime to the sentence did not become clear until several hundred milliseconds after the sentence onset, potentially limiting its influence. While English primes may have remained semantically active and supported early integration, the Spanish primes might have been suppressed too early during the switch to yield a measurable benefit. Future designs could address these limitations by embedding Spanish primes within the sentence itself. For example, a sentence-internal code-switch embedded in a biasing context could better approximate naturalistic switching and maintain semantic continuity. Such an approach may enhance the prime’s effectiveness in activating the appropriate semantic network, ultimately facilitating the interpretation of the figurative target.

## Conclusion

The present study demonstrates that bilingual language processing is influenced by both priming effects and language context, as reflected in behavioral and ERP measures. While behavioral data showed no priming or language effects, ERP results revealed distinct patterns in the N400 and LPC time windows, emphasizing the interplay between early semantic mismatch detection and later integrative processing. Larger N400 amplitudes for anomalous sentences, particularly in the within-language priming condition, underscore the heightened sensitivity of shared linguistic contexts in detecting semantic anomalies. In contrast, novel metaphors and novel similes elicited more pronounced LPC responses, reflecting the additional cognitive effort required for meaning integration and reanalysis. The absence of robust priming effects in the between-language condition and the impact of prime type highlight the complexities of bilingual language processing, including the challenges posed by code-switching and asymmetric language dominance. Together, these findings illuminate the dynamic processes underlying bilingual metaphor comprehension, emphasizing the critical roles of context, priming, and sentence type in shaping neural responses to figurative and anomalous language.

## Data Availability

The data and codes are available at https://osf.io/vkag6/.

## Footnotes

1 We also performed post-hoc analyses using ERP data baseline-corrected to the 0–200 ms interval. These analyses yielded trends consistent with those reported in the main manuscript. Detailed results are available in the OSF repository.
